# Towards interruption of schistosomiasis transmission in sub-Saharan Africa: developing an appropriate environmental surveillance framework to guide and to support ‘end game’ interventions

**DOI:** 10.1186/s40249-016-0215-9

**Published:** 2017-01-14

**Authors:** J. Russell Stothard, Suzy J. Campbell, Mike Y. Osei-Atweneboana, Timothy Durant, Michelle C. Stanton, Nana-Kwadwo Biritwum, David Rollinson, Dieudonné R. Eloundou Ombede, Louis-Albert Tchuem-Tchuenté

**Affiliations:** 1 Department of Parasitology, Liverpool School of Tropical Medicine, Pembroke Place, Liverpool, L3 5QA UK; 2 Department of Environmental Biology and Health, Council for Scientific and Industrial Research-Water Research Insitute, P.O. Box M 32, Accra, Ghana; 3 Neglected Tropical Diseases Programme, Ghana Health Services, Accra, Ghana; 4 Department of Life Sciences; Natural History Museum, Cromwell Road, London, SW7 5BD UK; 5 Centre for Schistosomiasis and Parasitology, Yaoundé, Cameroon; 6 Laboratory of Parasitology and Ecology, Faculty of Sciences, University of Yaoundé I, Yaoundé, Cameroon; 7 National Programme for the Control of Schistosomiasis and Intestinal Helminthiasis, Ministry of Public Health, Yaoundé, Cameroon

**Keywords:** Environmental monitoring, Freshwater snails, eDNA, WASH, Zoonosis

## Abstract

Schistosomiasis is a waterborne parasitic disease in sub-Saharan Africa, particularly common in rural populations living in impoverished conditions. With the scale-up of preventive chemotherapy, national campaigns will transition from morbidity- to transmission-focused interventions thus formal investigation of actual or expected declines in environmental transmission is needed as ‘end game’ scenarios arise. Surprisingly, there are no international or national guidelines to do so in sub-Saharan Africa. Our article therefore provides an introduction to key practicalities and pitfalls in the development of an appropriate environmental surveillance framework. In this context, we discuss how strategies need to be adapted and tailored to the local level to better guide and support future interventions through this transition. As detection of egg-patent infection in people becomes rare, careful sampling of schistosome larvae in freshwater and in aquatic snails with robust species-specific DNA assays will be required. Appropriate metrics, derived from observed prevalence(s) as compared with predetermined thresholds, could each provide a clearer insight into contamination- and exposure-related dynamics. Application could be twofold, first to certify areas currently free from schistosomiasis transmission or second to red-flag recalcitrant locations where extra effort or alternative interventions are needed.

**Electronic supplementary material:**

The online version of this article (doi:10.1186/s40249-016-0215-9) contains supplementary material, which is available to authorized users.

## Multilingual abstract

Please see Additional file 1 for translations of the abstract into the six official working languages of the United Nations.

## Introduction

In sub-Saharan Africa, schistosomiasis is a waterborne parasitic disease of medical and veterinary importance particularly in impoverished, rural communities with limited access to safe water and adequate sanitation [1]. As many other trematodes, schistosomes have an intricate lifecycle involving two free-living motile larval stages, a ciliated miracidium and a birfurcate cercaria. Each stage resides in freshwater, both are short-lived being lecithotropic (non-feeding), but are exquisitely adapted to facilitate parasite transmission, by per-cutaneous routes, from vertebrate to intermediate snail host and *vice versa* [2]. This evolutionary specialisation has led to striking differences in the morphology, physiology and behaviour of the miracidium and cercaria, respectively [3, 4]. Although each stage is just visible to the naked eye, under the microscope they are so radically different in form and function that piecing them together within a coherent lifecycle was a major scientific break-through just over one hundred years ago [5]. Elucidation of the lifecycle revealed vulnerabilities and identified suitable attack points to control this formidable foe.

In terms of contemporary control of schistosomiasis in sub-Saharan Africa, preventive chemotherapy (PC) campaigns implementing mass drug administration (MDA) of praziquantel (PZQ), a broad spectrum anthelminthic, are the foundation of several national control programmes [6]. Each year, millions of school-aged children receive treatment with donated PZQ [7, 8], the only available schistosomicidal drug [9, 10]. Looking to the future, treatment targets in the WHO 2020 Roadmap encourage further scale-up of PC campaigns but despite desirable features, drawbacks of MDA include the inactivity of PZQ against immature worms, the poor cure rates associated with singe treatments, the inability of treatment to guard against re-infection and the challenge of maintaining adequate treatment coverage in currently targeted groups [9, 11–15]. Extra efforts to maximise the impact of PC are well-discussed and ideally should be set within an integrated control strategy inclusive of: water, sanitation and hygiene (WASH) interventions, health education with behavioural change, environmental modification and snail control with focal mollusciciding [15–18], as highlighted by the World Health Assembly (WHA) in resolution WHA65.21. Whilst there are challenges ahead [13, 19], there is optimism grounded on epidemiological evidence and theory that elimination of schistosomiasis transmission in certain settings is achievable [14, 20–22]. Progress towards elimination is outlined in the WHO 2020 Roadmap and as campaigns transition from morbidity- to transmission-related control, formal investigation of environmental transmission is required [6]. Surprisingly, there are no international or national guidelines to do so in sub-Sahara Africa. The WHA65.21 resolution called on the WHO to prepare guidance for Member States towards the elimination of transmission, to establish procedures for the confirmation of the interruption of transmission, and to support countries with post-elimination surveillance to prevent reintroduction of transmission. Collectively, these can be considered as interventions themselves from the ‘end game’ perspective.

## Transmission in the aquatic landscape

**Fig. 1:**
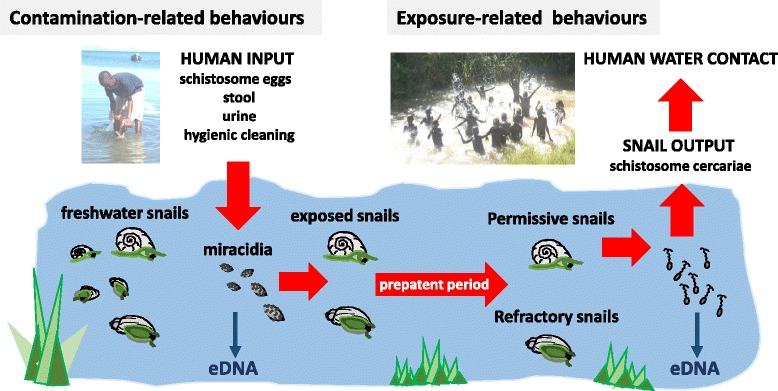
Key environmental aspects in schistosome transmission as framed by contamination- and exposure- related behaviours. Schistosome eggs can be introduced to freshwater by any infected vertebrate host, in this instance a mother and her pre-school-aged child (*who are not targetted in PC campaigns*) are depicted. After maturation in keystone species of freshwater snail hosts, schistosome cercariae are released often in copious numbers, which have a potential to infect any demographic group, like the school-aged children in the image depicting exposure-related behaviours (*who are the current target of PC campaigns*). Each day the emergence, death and decay of the larval stages contributes to aquatic planktonic assemblage and environmental (e) DNA components. Only those aquatic habitats that contain snails patently shedding cercariae pose a potential or actual threat to human health

Pertinent features of schistosomes in the aquatic landscape are shown in Fig. 1. In principle, aspects of environmental transmission can be broken down into two sequential and partially correlated components, contamination- and exposure-related inputs and outputs. Each aspect is inherently dynamic, ranging from singularly rare events, sporadic both in time and space, to almost continuous daily processes [17, 23, 24]. A rather paradoxical feature of the schistosome lifecycle, without any intervention, is that each day innumerable mass mortalities of the larval stages occur [24, 25]. This seemingly unwise daily sacrifice in biomass is, however, an evolved response to ensure successful transmission [26]. Both larval stages are non-feeding thus the vast majority of miracidia entering (hatched from eggs) and cercariae exiting (released from snails) simply fail to find a suitable host and therefore die [25]. Their lives are indeed ephemeral, their decay is an overlooked feature within planktonic assemblages and associated aquatic food webs [27].

When no zoonotic transmission is considered and only the human populace examined, the discrimination between contamination- and exposure-related events can be problematic. Those currently infected within the community can be further infected still, as additional exposure-related events take place. At the individual level, although distinction of first exposure is cardinal, classification of subsequent water contact activities becomes blurred for the balance between contamination-versus exposure-related events oscillates by location and in temporal pattern. A good example is provided by the depicted mother and child pair (see Fig. 1), a common sight along water body margins in sub-Saharan Africa. Here, the young child is at clear risk of first exposure but, once patently infected, will later excrete eggs to play an increasing role in contamination [28, 29]. Furthermore, it only takes one infected person to contaminate a water body to later create sufficient risk of exposure to others who enter later.

The important role of the younger children in contamination is especially true, if continued to be bathed in this manner, owing to more indiscriminate urination and defecation practices, and not forgetting that eggs of *Schistosoma mansoni* adhering to peri-anal areas [30] may be immersed, detach and hatch in extremely close proximity to freshwater snails. Increased micturition is associated with *Schistosoma haematobium* infection of the bladder and furthermore, entry into cool water typically stimulates a desire to urinate, often alleviating micturition pain. By contrast, the mother is also at risk of exposure, like many times before, but her role in contamination is mediated more by her child-bathing behaviour perhaps rather than her personal water contact activities and toileting. Nonetheless, each has a tangible potential to contaminate until a curative dose of PZQ is each received respectively. This points towards an immediate treatment gap in transmission control with PC as MDA with PZQ is primarily targeted to school-aged children alone [28]. This may result in insufficient targeting of treatments to those who maintain transmission, hence there is an imperative to expand access of PZQ to all members of the community who are infected and block contamination-inputs as best as possible if interruption of transmission is to be achieved. For example, the implementation research consortium of COU**NTD**OWN is specifically investigating new ways to expand access of treatment to these currently overlooked groups [5].

Other factors that influence direct egg-inputs are aspects of the shoreline location such as ease of access or the frequency of contacts, for example, washing in private or in communal areas, together with other contamination-related activities that vary by age, gender and cultural practices. Furthermore, indirect inputs of eggs from other sources such as those from overflowing latrines or that washed-in by rain, should not be ignored [24]. Other peculiar contamination-related activities include the purposeful spreading of human or animal faecal material by fisher folk used as fish attractants. Without a holistic oversight of all inputs, it is evident that any intervention, no matter how well executed, needs to consider all sources of contamination, else it may be mitigated by local confounders. Even though the use of an index of contamination has been proposed, it has not been widely used owing to site-specific heterogeneities [31]. While many aspects of this contamination-related landscape remain enigmatic there is a pressing need to better quantify these dynamics to ensure that this environmental component is not ignored and sensible criteria for elimination are developed [13, 14].

### Success by larval saturation

Only the tiny fraction of miracidia and cercariae that transition successfully between hosts could be considered successful, as some element of chance has favoured their progression, but it is by sufficiently saturating the environment that these rather unlikely transmission events become statistically inevitable [24, 32]. Furthermore, evolved subtleties in the chemotaxis and chronobiology of the larval stages favour the odds of transmission and fine-tune other life history traits, honed by natural selection for eons. Once inside the body of the next host, the schistosome undergoes totally different morphogenetic pathways with variable fecundities and lifespans. There are many unique and remarkable adaptations within the human body equipping the schistosome to live for many years, and in some cases decades.

On the other hand, once inside a permissive freshwater snail host, a single miracidia (which is either male or female) has a much shorter lifespan, not much more than a year, and undergoes rapid transformation shedding its cilia plates, later becoming a primary sporocyst. This then undergoes asexual reproduction dividing and growing in number into daughter sporocysts thence undergoing cercariogenesis releasing numerous cercariae. This pertinent feature of the lifecycle means that any sole analysis of miracidia as detected in water can only be a partial indicator or predictor of future exposure-related risk. Depending on the species of schistosome, this incubation or pre-patent period, can be as short as 3–4 weeks or be partially arrested taking up to several months to complete [33–35]. Snails can also be infected by more than one miracidium simultaneously and cross-species competitive antagonisms with other trematodes living in the same space within the snail are known [36]. Snails shedding cercariae can live up to several months, or during dry seasons aestivate, expanding the timeframe for exposure-related risks. One cannot over-estimate the population boost that occurs from only a handful of miracidia once inside a population of permissive snail hosts which then makes this habitat a dangerous place for exposure.

**Fig. 2:**
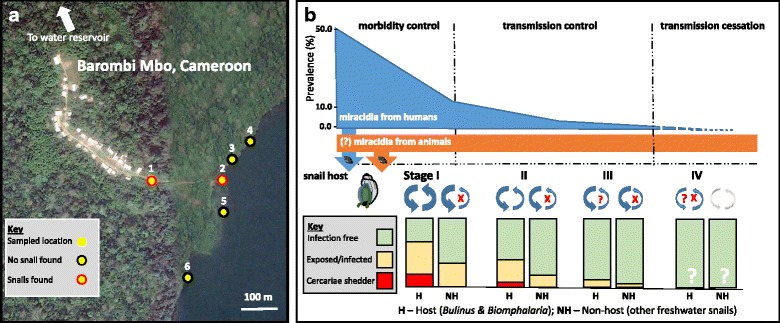
Key environmental aspects in schistosome transmission as framed by contamination- and exposure- related behaviours. **a** Image of Barombi Mbo, South-West Cameroon a small linear village recently sampled in May 2016 during a conjoint parastiological and malacology survey, finding the prevalence of egg-patent *S. haematobium* infection < 10%. Snails were searched for at six collecting locations (sites 1–6), only *B. forskalii* and *B. truncatus* were found at sites 1 and 2, with an average daily collection at each site inspection of 11 and 57 snails over a three day period, respectively. The survey highlights the small-scale heterogenities typical of schistosomiasis. **b** A schematic of the three phased progress of interventions from morbidity to transmission control then interuption of transmission, as prevalence of egg-patent infection declines as indicated by the blue section. At the sametime the miracidial input will likely concomittantly decline into the local snail fauna, in host (H) or non-host (NH) snails, respectively. Contrary to host snails, non-host snails do not produce cercariae hence play no later role in exposure-related transmission. Measuring and comparing the prevalence of schistosome DNA in H and NH species could provide information in the context of contamination-related and exposure-related measures at different stages during this transition. Conceptually, there should always be additional H snails that are patently (stage II) or pre-patently (stage III) infected and carry schistosome infections. Note that as the human miracidial input declines zoonotic sources may be more obvious and the need for species- and population-specific schistosome probes becomes essential

A central feature of the snail-schistosome interaction is differential host-parasite compatibility [37, 38]. Whilst a miracidium may successfully locate and penetrate, it may eventually fail to develop through all intra-molluscan stages owing to the snail’s internal defence system (IDS). There is a complicated evolutionary arms race between snail and schistosome in terms of immunity and population biology, a process of adaptation and counter-adaptation. Moreover, snail-schistosome evolution has been ongoing time immemorial and since miracidia will penetrate all freshwater snails it is possible to find evidence of their presence, by molecular detection methods, in non-host species before their components are removed or absorbed by the snail’s IDS [37, 38]. This residual phenomenon can be used as a pertinent feature in transmission surveillance as discussed below (see Fig. 2b).

### The importance of keystone snail species

In terms of ecology, any compatible snail species in which the schistosome can develop through to cercariogenesis can be considered a keystone species [23]. Without the presence of such keystone species, schistosomes would be unable to colonise any freshwater habitat either lentic or lotic in character. Therefore the geographical distribution of permissive *Bulinus* or *Biomphalaria* species provides a strong location-specific filter that restricts the effective exposure-related zone of *S. haematobium* and *S. mansoni*, respectively [39]. The broader distribution of snail species is largely determined by ecological factors at the macro-level, e.g. ecozone & climate, as well as *in situ* evolution, e.g. lake specific fauna [40, 41]. At the micro-level, however, other largely stochastic processes influence local distributions and snail population dynamics, e.g. dispersal & colonisation processes etc. [42–44]. In terms of life history ecology, all freshwater pulmonates are exemplars of r-species strategists (i.e. adapted to unstable environments, population density independent mechanisms) whilst their vertebrate hosts are K-species (i.e. adapted to stable environments, population density dependant mechanisms). Cercariae have evolved to bridge across this contrasting ecology of host populations, for they are powerfully adapted such that even a transient exposure is sufficient to gain entry and infection. People are much longer-lived, more peripatetic than snails, so it is by their entering into these aquatic zones that allows the schistosome to transfer from location to location. A good example, are the newly identified foci of autochthonous transmission on Corsica [45].

### Success by focalisation and spatial autocorrelation

Once again, exposure-related events that seem implausible by chance are profoundly inevitable given the copious numbers of cercariae released each day. Even though snails may be found in deeper water, for example in Lake Victoria up to 40 m in depth [46], at a micro-spatial level, infected snails typically fringe water-edge margins [46]. This is usually at entry points or other water-contact sites, where contamination-related events have taken place previously, so there is an inevitable spatial autocorrelation. Moreover, both *Bulinus* and *Biomphalaria* thrive in the aquatic landscape created or perturbed by mankind [44, 46–48]. This can be as early colonisers of irrigation schemes and water impoundment measures; in anoxic areas polluted by poor sanitation, laying numerous egg masses on discarded plastic and materials or by attaining high population numbers in areas depleted of molluscivorous fish by over-harvesting [49, 50].

It is these coalescence points of snail, human and schistosome interactions that cause the well-known focality of schistosomiasis and it is here where an environmental surveillance framework is most needed that measures schistosomiasis transmission as correctly as possible [14]. Put simply, all that is needed is a thorough and consistent sampling methodology, sufficiently robust to micro-spatial and seasonal temporal fluctuations. This is outlined in concept in Fig. 2 where the interplay of infections in people and snails is envisaged.

## On environmental sampling: practicalities and pitfalls

Once keystone species of snail were identified and larval stages of the schistosome could be recognised, a rich literature grew describing many basic aspects of the environmental biology of the schistosome [46, 51]. This largely drew upon studies in medical malacology, cercariometry and prospective epidemiology in the use of sentinel animals, such as laboratory-bred snails or rodents deployed in aquatic cages [52–58]. It is outside the scope of this paper to review this extensive literature comprehensively, only to suffice that each method had both positive and negative qualities [14]. A pervasive thread throughout has been arduous and disjointed sampling frames, ambiguities in host and parasite taxonomy and unreliable identification/detection with assays of poor sensitivity and specificity. With the presence of keystone species, it seems obvious that medical malacology should be an important component within any surveillance framework, much as the study of medically important mosquitoes is essential for malariology, however medical malacological studies in sub-Saharan Africa have waned and there is a recognised need for capacity building in this discipline [59].

This decline was largely due to abandonment of snail control with chemical molluscicides, downgrading the importance of snail identification, for Bayluscide® kills all snails [60], and an inability to develop field-based methods that accurately identified permissive host populations of *Bulinus* and *Biomphalaria*. However, well-described field-based methodologies were developed to monitor the ecology of snail populations [39]. This was primarily for application and optimisation of molluscicides specifically to keep host snail populations as small as possible [60, 61], and where successful has had a major public health impact [18], but sadly did little to quantify precisely the environmental epidemiology of schistosomiasis within a coherent framework. With the introduction of DNA profiling techniques, foremost with PCR, several problems in identification of snail and schistosome have been overcome, revitalising transmission biology studies, and opened a new vista on environmental surveillance [62–67].

### Highlighting significant transmission foci

A good example can be found in the resolution of the transmission biology of *S. haematobium* in Zanzibar which helped to focus efforts on those habitats holding *Bulinus globosus* and not *Bulinus nasutus* [68, 69]. This became a central tenet of the Zanzibar Elimination of Schistosomiasis Transmission (ZEST) project, for *B. nasutus* is a refractory host there [70, 71]. Others include schistosome-snail investigations around Lake Victoria. More broadly, the application of DNA-based assays in medical malacology [72] also coincided with the rise in DNA diagnostic assays [19], such as real-time PCR approaches with TaqMan® probes, for medical diagnostics [73, 74]. It is precisely that these medical diagnostic assays have become accepted, standardised and largely routine, that they can be used to spur on interests in environmental surveillance of schistosomes by inspection of water filtrates, environmental samples as well as in field-caught or sentinel snails [65, 66], see Table 1.

**Table 1:** Aspects of contamination- and exposure-related themes and outcomes

Aspect	Activity themes	Outcome indicators
	*Schistosome species of medical or veterinary significance, or of both?*	*Qualitative/quantitative, at what reporting scale - local or national?*
Contamination	Collection of host and non-host snails -*environmental variables (altitude, habitat type, limnology, seasonality etc.)* -*molecular detection of infection*	Snails present, abundance -*elevation, lentic or loctic, water temperature, pH, conductivity, season* -*%age thresholds (H:NH* ^*b*^ *)*
	Use of briefly deployed sentinel snails -*molecular detection of infection*	H snails (& NH if present) -*%age thresholds (H:NH)*
	Detection of eDNA^a^ -*molecular detection of schistosomes*	Schistosomes present -*schistosome species*
Exposure	Cercariometry -*molecular detection of schistosomes*	Schistosomes present - s*chistosome species*
	Collection of host snails -*identification of infection by microscopy* -*molecular detection of infection* -*molecular identification of cercariae*	Snails present, abundance -*patent schistosomes, chronobiology* * - %age thresholds (H)* * - schistosome species*
	Use of briefly deployed sentinel rodents -*non-invasive molecular diagnostics/autopsy*	Schistosomes present * - schistosome species*
	Detection of eDNA^a^ -*molecular detection of schistosomes*	Schistosomes present * - schistosome species*

^a^it is not possible to differentiate between miracidia and cercariae by current DNA methods, even with those that can currently differentiate larvae by epigenetic methylations, thus contamination- and exposure-related events are confounded

^b^H- host snail species (known to be permissive by finding natural infections or by experimental challenge), NH – non-host snail species (refractory in natural or by experimental challenge)

Several pioneering molecular studies have shown that many other species of freshwater snail carry evidence of schistosome infection than traditional methods have inferred previously [75–78]. Another important technical development has been the use of FTA card sampling methods which has enabled convenient storage of schistosome eggs, miracidia and cercariae for more precise genotyping of schistosome populations and species [62, 79–81]. This has revealed some intriguing population dynamics in people and snails, as well as, unexpected findings of cross-specific hybrids within the *S. haematobium* group, as encountered in Senegal and Niger [82], and the newly identified foci in the Cavu River, Corsica [83].

### On rational sample sizes and collecting sites

Even with robust DNA tools and techniques, two key epidemiological questions need to be resolved in developing an appropriate environmental surveillance framework. First how many snails (inclusive of their type and size) need to be collected at each site to accept or reject an infection prevalence below a predetermined threshold with confidence? Second, how many collection sites need to be considered, how should they be selected, and how often inspected, to gain a realistic appraisal in reduction in or cessation of transmission? Answers to each question have to be logically consistent as well as feasible in implementation.

**Fig. 3:**
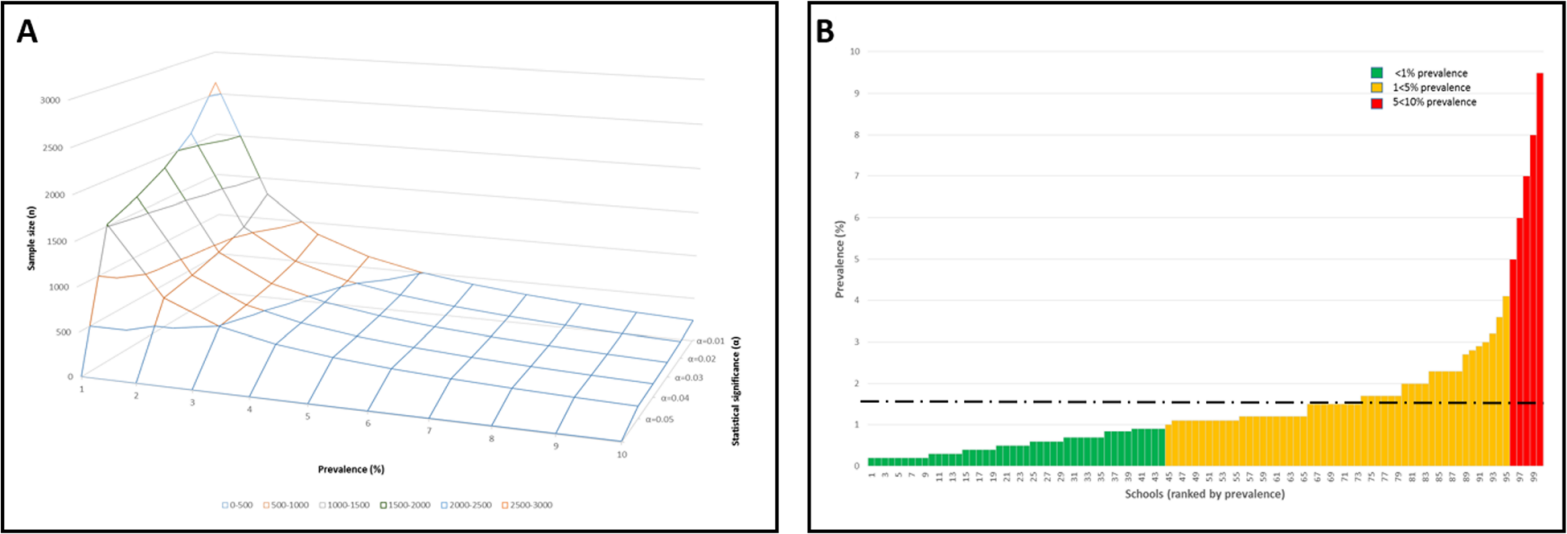
**a** Plot of sample size calculations for low prevalence (10% and less) settings, demonstrating the effect on sample size of reducing prevalence towards 1%, and of increasing the statistical significance (α). In principle, this hypothetical surface could derive from any diagnostic. However, as more sensitive diagnostics are each applied, the surface shape will remain similar only now with a raised offset, as previously ‘missed’ infections are subsequently detected. Note that even at assumed 10% prevalence of *Schistosoma*-infected snails, sample sizes for any level of significance of α = 0.05 or more are already between 140 and 240 snails; this increases as prevalence reduces and as more precision and statistical significance are applied, to levels that are laregly impractical (1500–2700 snails). Formula used is: $n={\left({Z}_{\frac{a}{2}}\right)}^2\rho \left(1-\rho \right)/{d}^2$, where: n = sample size, *p* = estimated prevalence, *d* = precision of the estimate (with the assumption that *d* = 0.5*p given low prevalence setting), Zα/2 = the Z-statistic associated with the statistical significance α/2 (Z-statistic adjusted for each of α = 0.05 to α = 0.01) [94]. **b** Plot of prevalence of schistosomiasis across 100 schools (mean prevalence of 1.5%), ranked in ascending order according to the well-known pattern of overdispersion or focalisation. It may be proportionately easier to find infected snails in water contact sites surrounding those schools in red, while it will be harder around those schools in green. A flexible sample size criteria seems sensible where more geographical attention is given to those habitats in the vicinity of schools in red rather than around schools in green

Responses to the first question can rely in part on guidance from classic epidemiology in basic sample size calculation and theory. Typical of elimination settings, as any infection becomes rare, proving a reduction in or absence of transmission dramatically increases required sample sizes and basic sample size formulae are ill-equipped for surveillance of disease at very low prevalence or incidence [19]. The four main determinants of sample size are (i) the magnitude of effect; (ii) the variation (standard error) of the study outcome; (iii) the confidence intervals; and (iv) study power. To detect a small effect size (i.e., prevalence of infection <10% by DNA assays), with high variation (standard error), and narrow confidence intervals and power (precision of the estimate), each determinant separately and cumulatively means that a large sample size is required (see Fig. 3a). This immediately places an operational strain and financial constraint on envisaged resources.

For rare prevalence outcomes, estimates need to be very precise. Increasing this precision, or increasing the statistical significance, will be required in sample size calculations; this itself requires judgement calls as there are no set ranges of precision specifically recommended for low-endemicity settings. However, sample size calculations increase so dramatically that they rapidly become unrealistic in terms of practical sampling. Thus, as detectable infection with schistosomes wane, an epidemiological ‘stalemate’ in surveillance will be reached for it is not feasible nor practical to sample regularly an entire population of snails [84]. Conceptually, while it is easier to prove something is there rather than is not, this tipping point in theory versus evidence needs to be found that leads to sufficient proof that an absence of transmission, either contamination- or exposure-related, can be confirmed. Mathematical models suggest that even modest influx of parasites can lead to (re-)initiation of transmission which may well go under the radar of current methods of field-sampling [85]. An unforeseen implication is that unless alternative statistical frameworks are developed and applied, not just in snails but also in people, arguably in the interim, it may be more cost-effective to simply continue with MDA without any investigation of transmission [86].

### Reconciling random processes and real natural history

The focality and highly skewed geographical distribution of schistosomiasis [87–90], illustrated in Fig. 3b, poses a significant challenge in addressing the second epidemiological question, the spatial selection and number of collection sites to be examined through time. Random sampling frames may be an epidemiological default, especially for diseases with poorly known aetiologies, with the underlying assumption that any sample must be representative of the broader population. Random sampling is a design-based sampling approach which has the goal of obtaining an estimate of a summary measure of the overall population e.g. prevalence. This is, however, not the most optimal spatial sampling framework if the goal is to explore and understand more about the spatial heterogeneities of the phenomenon under consideration. Therefore, a different approach needs to be considered to identify contamination- or exposure-related transmission events. Lattice-based spatial sampling approaches have been shown to be optimal where the goal is to predict the spatial distribution of an outcome but as transmission events become progressively sporadic and more will-o’-the-wisp-like in nature, the required fine-scale resolution of the lattice required to accurately predict transmission events might be too prohibitive for this approach to be feasible. In short, to inspect collection sites at random and ignore sites having prior suspicion or knowledge of contamination-like activities, would be logically perverse. Local ‘proof’ of the cessation of transmission can only be ascertained on a site-by-site basis. Schistosomiasis transmission in many areas will be seasonal and due account must be taken of climatic factors, transmission may be intense at certain time of the year and completely absent at others [91].

To develop a consistent sampling framework, any transmission site to be first assessed must be initially selected purposively, with some *a priori* consideration of local snail fauna and water contact with contamination- and exposure-related criteria, before any sub-sampling, whether random or spatially structured, is performed. Further exploration will then need to be undertaken to establish the extent of the spatial autocorrelation (if any) in the snail population, e.g. using semi-quantitative statistics and qualitative aspects [84, 90]. The inappropriateness of an entirely random spatial framework is clearly demonstrated with the data from Barombi Mbo (see Fig. 2a), whereby this approach would have missed either or both of the two freshwater sites in which *Bulinus* were later encountered. This clearly demonstrates a formal need to recognise and include local knowledge and understanding of site specific-heterogeneities within an adaptive sampling protocol rather than to overlook them.

## Towards an appropriate sampling framework

Advancing a transmission surveillance system for schistosomiasis based upon a default assumption of random processes and associated sampling strategies is flawed. Rather, sampling frameworks should be semi-structured to take into account the focal nature of this disease and the peculiarities of intermediate snail host distributions and dynamics. It is presently clear that further research is needed to focus dialogue and reach a useful consensus at either international or national levels.

To this end, there needs to be much better cross-talk and information exchange within the health sector to bridge the human-health and environmental-health divide. Historical data on actual or predicted snail distributions and abundance should not be ignored. This should be used to help guide general areas for more detailed investigations, concurrently with available point-prevalence maps of people to incriminate specific points of transmission. Focal site selection requires ground-truthing as a rapid assessment to confirm the presence of snail intermediate hosts, before more detailed sampling can be undertaken. To reduce the likelihood of sites being selected based on individual preference, the additional elements of local water chemistry and human water contact patterns should be considered. Monitoring human water contact behaviours in sites is an inherently important, but oft-ignored, feature for even with presence of snail intermediate hosts, there is likely to be little evidence of schistosomiasis transmission in water bodies that have negligible human contact patterns, notwithstanding zoonotic sources [79].

A key feature of any appropriate sampling framework is that it needs to be both feasible and implementable within realistic levels of resourcing. Despite acknowledged limitations, an advantage of purposive sampling is to better forecast and define key areas for scrutiny. An approach that will yield both semi-quantitative and qualitative informative data and quickly able to red-flag locations where progress has been poorer than expected. Using a combination of molecular-based approaches with traditional parasitological sampling methods in field-caught or sentinel snails is a powerful combination to reveal evidence of contamination- and exposure-related transmission, foremost for higher levels of DNA detected in host snails can differentiate those playing exposure- rather than contamination-related roles [80, 92]. Furthermore, there are no ethical restrictions on collecting or crushing snails, and samples can be readily transported in ethanol for DNA analyses undertaken in laboratories where molecular diagnostics are undertaken. Descriptive data on key characteristics of the local human population (e.g. community size, geographic spread, water contact activities and water contact points, disposal of wastewater, WASH infrastructure, etc.) should supplement the snail survey data and help to pinpoint if reduction in transmission has not been achieved and help to explain the reasons why.

Accuracy in disease measurement and detection will be crucial to control schistosomiasis transmission. The inverse relationship between statistical precision to detect disease and sample size in near-elimination settings, where resource trade-offs are unlikely to be possible, highlights unresolved issues around the accuracy and applicability of standard sample size formulae in these settings. The epidemiology obviously needs to be accompanied by very sensitive diagnostic testing, as in the absence of “proven” transmission cessation, risks of resurgence need to be appropriately estimated. The application of DNA screening of snails in very low prevalence settings, as a contamination-related indicator, is likely to be critical and needs to be further investigated. Looking further, extremely focussed active surveillance measures will be required to prevent re-establishment of transmission through sporadic or introduced peripatetic cases [14]. Such environmental strategies do not supplant the need to continue refining ‘end game’ schistosomiasis targets and surveillance in humans; rather they represent potentially low-cost strategies to contribute knowledge that could aid in the further targeting of resources to sites of greatest need. Our article represents a starting point for considering an environmental framework. Next steps will be to fully define and explore these issues, perhaps within recent bilateral initiatives [93], with the aim of developing operational protocols for future endorsement by the WHO-Geneva and WHO-AFRO at international and national levels.

## Conclusion

When developing an environmental surveillance framework able to certify areas free from schistosomiasis transmission differentiating between contamination- and exposure-related events is crucial. In so doing, greater attention can be placed on collection of the key samples and specimens, better tailoring local resources and negating confounding issues. Since each transmission landscape has unique features and dynamics through time, setting exacting and inflexible criteria is not advised. Establishing certain generic measures is, however, feasible such as observing an absence, or predetermined thresholds of prevalence, of schistosome DNA detected in snails. This could be measured and compared in field-caught host snails versus non-host snail species, and bolstered by deployment of sentinel snails of either host type. Taken together, this would be sufficient to provide testable criteria in the certification of interruption of environmental transmission or able to red-flag concerns in near interruption settings to intensify intervention efforts to synergise impact.

## Additional file

Additional file 1: Multilingual abstracts in the six official working languages of the United Nations. (PDF 635 kb)

## Abbreviations

IDS: Internal defence system
MDA: Mass drug administration
NTDs: Neglected tropical diseases
PC: Preventive chemotherapy
PZQ: Praziquantel
WASH: Water, sanitation and hygiene
