# Supplementary material for: Towards interruption of schistosomiasis transmission in sub-Saharan Africa: developing an appropriate environmental surveillance framework to guide and to support ‘end game’ interventions
Source: Infect Dis Poverty. 2017 Jan 14;6:10. doi: 10.1186/s40249-016-0215-9 (PMC5237522; doi:10.1186/s40249-016-0215-9)
Supplement: Additional file 1: — Multilingual abstracts in the six official working languages of the United Nations. (PDF 635 kb) [file 40249_2016_215_MOESM1_ESM.pdf]

Translation of the abstract into the six official working languages of the United Nations

نحو وقف انتقال مرض البلهارسيا في أفريقيا جنوب الصحراء الكبرى: تطوير إطار مراقبة بيئية مناسب لتوجيه ودعم المرحلة النهائية "للتدخل الطبي"

راسل ستوثارد، سوزي كامبل، مايك اوزي اتوينيبونا، تيموثي دورانت، مايكل ستانتون، نانا كاوادو بيريتاوم، ديفيد رولينسون، ديدون ايلاودو اومبيد، لويس البيرت تشويم تشينتي.

ملخص الدراسة
البلهارسيا هو مرض طفيلي تحمله المياه في أفريقيا جنوب الصحراء الكبرى. وهو شائع بين سكان المناطق الريفية الفقراء على وجه الخصوص. سينتقل تركيز الحملات الوطنية بسبب ارتفاع نسبة استخدام العلاج الكيميائي الوقائي من التدخل لعلاج المرض الى التدخل لمنع انتقال المرض. فمع ازدياد سيناريوهات المرحلة النهائية هناك حاجة للأبحاث الرسمية التي تدرس الإنحدار الفعلي والمتوقع لإنتقال المرض بفعل البيئة. ومن المثير للدهشة أنه لايوجد أي توجيهات دولية أو محلية للقيام بذلك في افريقيا جنوب الصحراء الكبرى. وبالتالي فإن مقالتنا هذه تقدم مدخلا إلى ماهو عملي وإلى العقبات الرئيسية في عملية تطوير إطار المراقبة البيئية المناسب. و ضمن هذا السياق فقد ناقشنا الحاجة لتغير الاستراتيجيات وتصميمها بما يتلائم مع المستوى المحلي من أجل دعم وإرشاد افضل للتدخل الطبي في المستقبل من خلال هذا التحول. وبعتبار أن الكشف عن إصابة البشر بالعدوى من خلال البيوض أصبح نادراً أصبح هناك حاجة لأخذ عينات من يرقات البلهارسيا في الماء العذب ومن قواقع الحلزونات المائية وإجراء فحص دقيق للحمض النووي (البصمة الوراثية) (DNA) الخاص بكل عينة. المقايس المناسبة المستمدة من الانتشار الملاحظ والتي تتم مقارنتها مع عتبات محددة سلفاً قد تقدم كل منها فهماً أوضح فيما يتعلق بالدينيماكية المتصلة بالتلوث والتعرض لهذه العدوى. أما عملية التطبيق فقد تكون ذات شقين. الأولى للمصادقة على المناطق الخالية حالياً من حلات إنتقال عدوى البلهارسيا أو الثانية وتتضمن المناطق الموبوؤة بشكل خطير و التي تحتاج إلى جهد إضافي أو إالى تدخلات بديلة.

Translated from English version into Arabic by Randa82, through

TRANSLATORS
WITHOUT BORDERS

在撒哈拉以南非洲阻断血吸虫病传播：建立适当环境监测框架，以指导和支持“终结游戏”干预

J. Russell Stothard, Suzy J. Campbell, Mike Y. Osei-Atweneboana, Timothy Durant, Michelle C. Stanton, Nana Kawado-Biritwum, David Rollinson, Dieudonné R. Eloundou Ombede, Louis-Albert Tchuem-Tchuenté

摘要
血吸虫病是的一种水源性寄生虫病，在撒哈拉以南非洲地区生活贫困的农村人群中尤其常见。随着预防性化疗的扩大，全国行动的干预重点从发病率转向传播。因此，当到达“终结戏”情景——血吸虫消除阶段时，需要对环境传播的实际或预期下降情况进行正式调查。但出乎意料的是，对此，撒哈拉以南非洲并无国际或国家指南。因此，本文介绍关于开发适当环境监测框架的重要实例和误区。在此背景下，我们讨论如何根据当地情况调整战略，以便今后更好地指导和支持干预措施。近来，随着检测人群虫卵感染越来越少，需要对淡水和水生钉螺中的血吸虫幼虫进行仔细取样，并进行稳定的种特异性 DNA 检测。从观测到的患病率（与预定阈值相比）导出的适当度量可更清楚地发现相关污染和暴露动力学特征。其作用可分为两个方面，首先是确认无血吸虫病传播区域，其次标注需要重点干预或需转变干预措施的顽固地区。

Translated from English version into Chinese by Jin Chen, edited by Pin Yang

CDC CHINESE CENTER FOR DISEASE CONTROL AND PREVENTION
NATIONAL INSTITUTE OF PARASITIC DISEASES

Vers une interruption de la transmission de la schistosomiase en Afrique subsaharienne: Développer une structure de surveillance environnementale pour orienter et appuyer les interventions « end game »

J. Russell Stothard, Suzy J. Campbell, Mike Y. Osei-Atweneboana, Timothy Durant, Michelle C. Stanton, Nana Kawado-Biritwum, David Rollinson, Dieudonné R. Eloundou Ombede, Louis-Albert Tchuem-Tchuenté

Résumé

La schistosomiase est une maladie parasitaire d'origine hydrique d'Afrique Subsaharienne, particulièrement répandue parmi les populations rurales qui vivent dans des conditions de grande pauvreté. Avec une plus grande échelle de la chimiothérapie préventive, les campagnes nationales passeront de la morbidité à des interventions focalisées sur la transmission, et donc une enquête officielle sur les baisses réelles ou attendues de la transmission environnementale s'avère nécessaire, à mesure que les scénarios 'end of game' apparaissent. De façon surprenante, il n'existe pas de directives internationales ou nationales pour faire cela en Afrique subsaharienne. Notre article fournit donc une introduction aux principaux aspects pratiques et écueils dans la création d'une structure de surveillance environnementale adaptée. Dans ce contexte, nous discutons sur les façons dont les stratégies doivent être adaptées et ajustées au niveau local pour mieux orienter et appuyer les futures interventions tout au long de cette transition. Etant donné que la détection d'une infection latente par les œufs dans la population devient rare, un échantillonnage rigoureux avec de solides tests ADN plus spécifiques des larves de schistosome dans de l'eau douce et des escargots aquatiques seront nécessaires. Des mesures appropriées, provenant de prévalence(s) observée(s) par rapport à des seuils prédéterminés, pourraient fournir chacune un meilleur aperçu de la contamination – et d'une dynamique associée à une exposition. L'application pourrait être double, premièrement pour certifier les régions réellement exemptes de transmission de la schistosomiase, ou deuxièmement, pour signaler les endroits récalcitrants, dans lesquels des efforts supplémentaires ou des interventions alternatives sont nécessaires.

Translated from English version into French by Ode Laforge, through

TRANSLATORS
WITHOUT BORDERS

Прекращение процесса распространения шистосомоза в странах Африки, расположенных к югу от Сахары: разработка соответствующих мер экологического надзора для контроля и поддержания окончательного воздействия

Дж. Рассел Стотхард (J. Russell Stothard), Сузи Дж. Кэмпбелл (Suzy J. Campbell), Майк Ю. Осеи-Атвенебоана (Mike Y. Osei-Atweneboana), Тимоти Дюрант (Timothy Durant), Мишель С. Стэнтон (Michelle C. Stanton), Нана Кавадо-Биритвум (Nana Kawado-Biritwum), Дэвид Роллинсон (David Rollinson), Дьедоннэ Р. Элунду Омбэдэ (Dieudonné R. Eloundou Ombede), Луи-Альберт Тчуэм-Тчуэнтэ (Louis-Albert Tchuem-Tchuenté)

Реферат статьи

Шистосомоз является заболеванием, вызываемым паразитами, передающимися через воду. Заболевание распространено в странах Африки, расположенных к югу от Сахары, и особенно характерно для сельского населения, проживающего в условиях нищеты. Благодаря более широкому применению профилактической фармакотерапии национальные кампании перейдут от воздействия, сосредоточенного на заболеваемости, к воздействию, сосредоточенному на распространении болезни. Таким образом, официальное исследование фактического или ожидаемого снижения уровня распространения через окружающую среду является необходимым. Удивительно то, что в африканских странах, расположенных к югу от Сахары, нет соответствующих международных или национальных рекомендаций. Поэтому наша статья представляет ключевые практические аспекты и ошибки в разработке соответствующих мер экологического надзора. В этом контексте мы обсудим, как следует адаптировать стратегии с учетом местного уровня. Целью этого являются более эффективные контроль и поддержание воздействия в будущем с помощью упомянутого перехода. Поскольку обнаружение явной инфекции у людей происходит редко, потребуется тщательная проверка пресной воды и водных улиток для выявления личинок шистосом с помощью надежной ДНК-диагностики. Соответствующие показатели, производные от наблюдаемой заболеваемости по сравнению с предварительно определенными пороговыми показателями, могут обеспечить более четкое понимание динамики загрязнения и воздействия. Применение может быть повторным, во-первых, для определения территорий, где шистосомоз в настоящее время не распространяется, и во-вторых, для выделения местностей, где необходимы дополнительные усилия или альтернативные меры воздействия.

Translated from English version into Russian by Larysa K., through

TRANSLATORS
WITHOUT BORDERS

Hacia la interrupción de la transmisión de la esquistosomiasis en el África subsahariana: Elaboración de un marco de vigilancia ambiental adecuado a fin de guiar y apoyar las intervenciones en la etapa final

J. Russell Stothard, Suzy J. Campbell, Mike Y. Osei-Atweneboana, Timothy Durant, Michelle C. Stanton, Nana Kawado-Biritwum, David Rollinson, Dieudonné R. Eloundou Ombede, Louis-Albert Tchuem-Tchuenté

Resumen
La esquistosomiasis es una enfermedad parasitaria de transmisión hídrica en el África subsahariana, particularmente generalizada en las poblaciones rurales que viven en condiciones de pobreza. Con la ampliación de la quimioterapia preventiva, las campañas nacionales pasarán de las intervenciones centradas en la morbilidad a las centradas en la transmisión, por lo que se necesitarán investigaciones formales sobre la disminución real o prevista de la transmisión ambiental a medida que surjan situaciones de etapa final o "final del juego". Sorprendentemente, no existen directrices internacionales ni nacionales para llevarlo a cabo en el África subsahariana. Por ello, nuestro artículo ofrece una introducción a los aspectos prácticos clave y los problemas en el desarrollo de un marco de vigilancia ambiental adecuado. En este contexto, deliberamos sobre cómo adaptar las estrategias y ajustarlas al ámbito local para guiar y apoyar mejor las futuras intervenciones durante dicha transición. A medida que la detección de la infección patente en personas se hace menos frecuente, será necesario un muestreo cuidadoso de las larvas de esquistosomas en agua dulce y en caracoles acuáticos con análisis consistentes de ADN específico de la especie. Las mediciones apropiadas, derivadas de la(s) prevalencia(s) observada(s) en comparación con umbrales predeterminados, podrían proporcionar una visión más clara de la dinámica relacionada con la contaminación y la exposición. La aplicación podría ser doble: en primer lugar, para certificar qué áreas no presentan actualmente transmisión de esquistosomiasis; o, en segundo lugar, para marcar los emplazamientos refractarios, en las que se necesitan esfuerzos adicionales o intervenciones alternativas.

Translated from English version into Spanish by Elisa Martinez-Aznar, through

TRANSLATORS
WITHOUT BORDERS
